# Monitoring the Changes in Heat Transfer and Water Evaporation of French Fries during Frying to Analyze Its Oil Uptake and Quality

**DOI:** 10.3390/foods11213473

**Published:** 2022-11-01

**Authors:** Ying Li, Qi Guo, Kaili Wang, Maheshati Nverjiang, Kairong Wu, Xu Wang, Xiufang Xia

**Affiliations:** College of Food Science, Northeast Agricultural University, Harbin 150030, China

**Keywords:** French fries, heat transfer, water loss kinetic, oil uptake kinetic, quality

## Abstract

The effect of frying temperature on heat transfer, water loss kinetic, oil uptake kinetic, and quality of French fries was evaluated. With increasing frying temperature, the core temperature of fries increased, and the Biot number and heat transfer coefficient (h) first decreased and then increased significantly (*p* < 0.05). The water loss rate (k_w_) and water effective diffusion of fries increased with the increasing frying temperature. The k_w_ of fries fried at 150–190 °C were 0.2391, 0.2414, 0.3205, 0.3998, and 0.3931, respectively. The oil uptake rate (k_o_) first increased and then decreased with increasing frying temperature, and the k_o_ of samples fried at 150–190 °C were 0.2691, 0.2564, 0.4764, 0.3387, and 0.2522, respectively. There were significant differences in the a*, L*, ΔE, and BI between fries with different temperatures (*p* < 0.05), while there was no significant difference in the b* (*p* > 0.05). The hardness and crispness of fries increased with increased frying temperature. The highest overall acceptability scores of fries were fried at 170 °C. Therefore, the changes in color, texture overall acceptability, and oil content were due to the Maillard reaction and the formation of porous structure, which was induced by h and water evaporation of fries when they changed.

## 1. Introduction

French fries are a casual fried snack with golden color, crispy texture, and attractive flavor, which are popular with consumers. However, the oil content of French fries is in the range of 30–50% [1]. Excessive intake of fried foods with high oil content may increase the risk of high blood pressure, obesity, heart diseases, diabetes, and so on [2]. Low-oil fried foods with excellent quality are expected by consumers and businesses, which drive the latest research trend.

Currently, the research of the fried foods’ oil content and quality focus on monitoring frying temperature and time [3], developing new frying technologies [4], using pretreatment [5], and so on. New frying and pretreatment technologies, such as microwave-assisted vacuum frying [6], electric field frying [7], and hot-air pre-drying [5], are cumbersome to operate, time-consuming, and high-cost. Frying temperature is the basic and critical parameter that influences the oil content and quality during frying. Liu, Tian, Zhang, and Fan [8] found that, as the frying temperature increased from 140 °C to 180 °C, the oil content of chips decreased by 14.7%.

There are three possible mechanisms to explain the oil absorption during frying, including water replacement (oil entered into the channels left by water evaporation), surfactant effect (the oil easily penetrated into food, due to the reduced interfacial tension between oil and food), and cooling effect (oil adhered to food’s surface was sucked into the cracks on the surface of food) [9]. The first two occur in the frying process, and the third occurs in the cooling phase after frying. Yu, Li, Ding, Hang, and Fan [10] studied that frying was a surface phenomenon, and the oil absorption behavior mainly occurred after frying. Li et al. [11] reported that there was a strongly positive correlation between the water and oil content during frying. The main mechanism of oil absorption in fried food is still controversial. In fact, the frying process involves heat transfer and moisture loss [10], which gave rise to starch gelatinization [12], Maillard reaction [13], protein denaturation [14], the formation of surface roughness [5], etc., which can affect the oil content and sensory attributes in fried foodstuffs. Sun et al. [6] reported that the frying temperature influenced the degree of Maillard reaction. Fanue et al. [15] studied the higher the frying temperature, the more energy transferred from frying medium to food. However, the studies for the mechanisms of oil uptake and quality changes by monitoring the dynamic changes of heat transfer and water evaporation were few.

Therefore, this study aimed to: (1) explore the effect of frying temperature on the heat transfer of fries during frying; (2) monitor the dynamic changes in water and oil content of fries; (3) establish the modeling of water loss and oil uptake kinetics of fries with different frying temperatures; (4) study the effect of frying temperature on the color, texture, and sensory evaluation of fries.

## 2. Materials and Methods

### 2.1. Samples Preparation

The frozen potato strips were obtained from Langweston Potato Industry Co., Ltd. (Inner Mongolia, China), cut into 5 × 1 × 1 strips, and stored at −18 °C. The oil used in the experiment was soybean oil, which was purchased from the local store (Harbin, China). The potato strips were fried in soybean oil at 150 °C, 160 °C, 170 °C, 180 °C, and 190 °C for 1–13 min using a thermostatically temperature-controlled fryer (DF25A, Guangzhou, China). After frying, the samples were removed and shaken in an aluminum basket to drain off for 30 s.

### 2.2. Core Temperature and Heat Transfer Coefficient (h)

The thermocouples were inserted into the geometric center of potato strips, and the sample was fixed in the middle of fryer and fried for 3 min. A digital multimeter was used to record the temperature in the center of fries from 0–3 min [15]. The Biot number (B_ih_) and heat transfer coefficient (h) was obtained from the temperature curves of French fries, which was calculated by Equations (1)–(3):

(1) $$Ln\;\frac{T\left(t\right)-T_{\infty }}{T_{0}-T_{\infty }}=lnA-2\mu_{1}{}^{2}\frac{\alpha t}{L^{2}}$$

(2) $$A=\left(\frac{2sin\mu_{1}}{\mu_{1}+sin\mu_{1}cos\mu_{1}}\right)2\cos\left(\mu_{1}\frac{x}{L}\right)\cos\left(\mu_{1}\frac{y}{L}\right)$$

(3) $$B_{ih}=\frac{hL}{k}=\mu_{1}tan\mu_{1}$$

Here, *T(t)*: temperature at t s (°C); *T_∞_*: temperature of frying oil (°C); *T*_0_: temperature of fries in the 0 min (°C); μ_1_: a parameter; α: thermal diffusivity (1.45 × 10^−7^ m^2^/s); *x* and *y*: location of temperature measurement in the infinite plates; *L*: half-thickness of potato strips (m); *B_ih_*: transfer heat Biot number; *h*: heat transfer coefficient (W/m^2^·K). The μ_1_ was calculated by the slope of curves of $Ln\;\frac{T\left(t\right)-T_{\infty }}{T_{0}-T_{\infty }}$ vs. time.

### 2.3. Water Content

The water content was measured by the method of AOAC (2000) [16]. The water content of fries was measured every 1 min. The water content was expressed in g/100g db.

### 2.4. Water Loss Kinetics of French Fries

The experiment data was obtained from the curves of water content vs. time. The water loss kinetics of French fries was expressed by Fick’s second law of diffusion (Equation (4)) [17].

(4) $$\frac{\partial }{\partial L}\left[D_{eff}\frac{\partial }{\partial L}\right]=\frac{\partial \left(M\right)}{\partial t}$$

Assuming the diffusivity and oil temperature was constant, and the shrinkage and external resistant of fries were negligible, the water loss rate (k_w_) of fries was calculated according to Equation (5).

(5) $$M_{r}=\frac{M-M_{e}}{M_{0}-M_{e}}=\frac{8}{\Pi }\sum_{n=1}^{\infty }(\frac{1}{(2n+1)2})exp\left[-{\left(2n+1\right)}^{2}\Pi^{2}\frac{D_{eff}t}{4L^{2}}\right]$$

For long frying time, Equation (5) could be simplified to Equation (6):

(6) $$M_{r}=\frac{M-M_{e}}{M_{0}-M_{e}}=\frac{8}{\Pi }\exp\left(-\frac{\Pi D_{eff}t}{4L^{2}}\right)=\frac{8}{\Pi }\exp\left(-k_{w}t\right)$$

Here, *M*: water content of fries at t min (g/100g, db); *M*_0_: initial water content of potato strips (g/100g, db); *M_e_*: equilibrium water content (g/100g, db); *M_r_*: water ratio (dimensionless); *L*: half-thickness of potato strips (m); *D_eff_*: water effective diffusivity (m^2^/min); *k_w_*: water loss rate (min^−1^).

The water effective diffusivity (*D_eff_*) was calculated from Equation (7):

(7) $$K_{w}=\frac{\Pi^{2}D_{eff}}{4L}$$

The activation energy of water loss in fries was calculated by Equation (8) [18]:

(8) $$k=k_{0}\exp\left(-\frac{E_{a}}{RT}\right)$$

where *k*: water loss rate; *k*_0_: frequency factor; *Ea*: activation energy; *R*: universal gas constant; *T*: frying temperature in Kelvin.

### 2.5. Oil Content

The oil contents of fries fried at different temperatures were determined every 1 min using Soxhlet extraction method. The oil content was expressed in g/100g db.

### 2.6. The Oil Uptake Kinetics

The oil uptake kinetic of fries was described by Equation (8) [18]:

(9) $$O_{r}=\frac{O-O_{e}}{O_{0}-O_{e}}=\exp\left(-k_{o}t\right)$$

Here, *O*: oil content of fries at t s (g/100g db); *O_e_*: equilibrium oil content (g/100g db); *O*_0_: initial oil content of fries; *O_r_*: oil ration; *k_o_*: oil uptake rate. When *t* = 0, the oil content was 8.9 ± 0.5 g/100g db.

The activation energy of water loss in fries was calculated by Equation (10):

(10) $$O_{e}=O_{1}\exp\left(-\frac{E_{a}}{RT}\right)$$

where *O*_1_: pre exponential factor; *E_a_*: activation energy of oil absorption.

### 2.7. Color

The lightness (L*), redness (a*), and yellowness (b*) values of French fries were measured using ZE-6000 colorimeter (Juki Corp, Tokyo, Japan) by the method of He et al. [19]. The color difference (Δ*E*) and browning index (*BI*) of fries were calculated by Equations (11)–(13):

(11) $$\Delta E=\sqrt{{\left(L^{*}-L_{0}{}^{*}\right)}^{2}+{\left(a^{*}-a_{0}{}^{*}\right)}^{2}+{\left(b^{*}-b_{0}{}^{*}\right)}^{2}}$$

(12) BI=(100×(x−0.31))/0.17

(13) $$x=\left(a^{*}+1.75L^{*}\right)/\left(5.645L^{*}+a_{0}{}^{*}-3.012b^{*}\right)$$

where *L*_0_*, *a*_0_*, and *b*_0_* are the color parameters of unfried samples, L*, a*, and b* are the color parameters of French fries.

### 2.8. Texture

The texture of French fries was measured by the puncture test using TA.XT Plus C texture analyzer (Stable Microsystems, UK), according to the method of Zhang et al. [20] and Li et al. [21], with some modifications. After frying, the fries were taken out of the fryer, and as the surface temperature of fries was cooled to 50 °C, the puncture test was conducted.

### 2.9. Sensory Evaluation

The samples were used for sensory analysis, including the French fries fried at 150 °C, 160 °C, 170 °C, 180 °C, and 190 °C. The samples were served to the assessors in plastic boxes labeled with three random numbers [22]. The groups of 20 trained assessors were divided into 2 groups of 10 members. The color, crispness, aroma, oiliness, and overall acceptability of fries were evaluated using a 9-point hedonic scale for each sensory attribution.

### 2.10. Statistical Analysis

All experiments were conducted at least three times. The data were provided with mean values ± standard error (SE) adopting a Student’s t-test by Statistic 8.0 software [23] (Analytical Software, St. Paul, MN, USA). The level of significance was set to *p* ≤ 0.05.

## 3. Results and Discussion

### 3.1. Core Temperature

**Figure 1:**
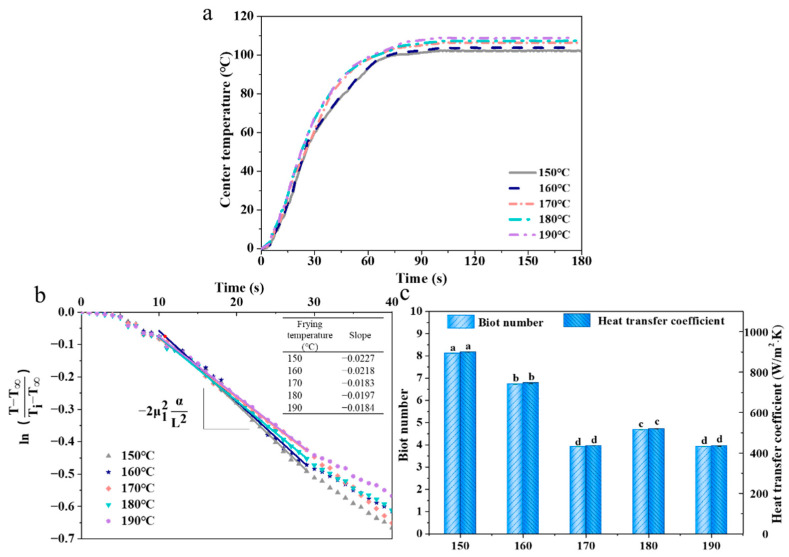
Effect of frying temperature on the center temperature (**a**), slope of linear sections of dimensionless temperature ratio vs. time plots (**b**), Biot number and heat transfer coefficient (**c**) in French fries. The results are the mean ± SE. Different letters (a–d) indicate significant differences (*p* ≤ 0.05).

As the frying time prolonged, the core temperatures of all samples rose first (<90 °C) and reached a plateau (>90 °C) and were finally in the range of 100–110 °C (Figure 1a). This result could be explained as follows: as the core temperature of French fries reached the boiling point of water, the energy transferred from the frying medium (frying oil) to the interior of fries was used for water evaporation, which led to less energy being used for the increase of the internal temperature [24]. Meanwhile, the rising rate of the center temperature in the fries increased when the frying temperature increased, which was attributed to the fact that the heat transfer and inward migration of water evaporation increased, allowing more energy to be used to increase the core temperature [25].

The B_ih_ and h values (Figure 1c), respectively, reflected the ratio of internal thermal resistance to external thermal resistance of the fries and effective heat transfer coefficient, which were calculated by slopes of linear sections of dimensionless temperature vs. time plots (Figure 1b). The slopes, B_ih_, and h values of the fries increased first and subsequently decreased when the temperature increased, and the minimum value was obtained at 170 °C, and there was no significant difference in B_ih_ and h values at 170 °C and 190 °C (*p* > 0.05). The reduction of slopes, B_ih_, and h values indicated that the heat transfer transferred from the frying oil to the interior of the fries was hindered, which was attributed to the reduced oil interfacial tension when water evaporated during frying, resulting in the formation of surface bubbles [26]. As the frying temperature increased above to 170 °C, the outside of fries formed a hard crust, which inhibited the water loss inside the fries [27].

### 3.2. Water Content and Kinetic Modeling of French Fries

#### 3.2.1. Water Content

**Figure 2:**
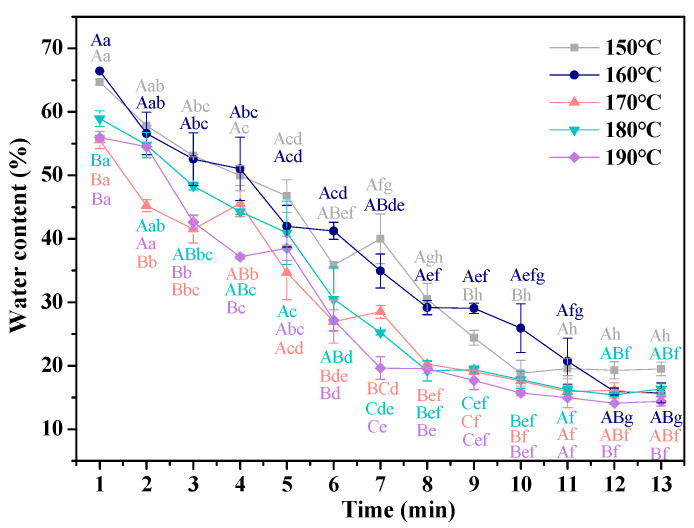
Effect of frying temperature and time on the water content of French fries. The results are the mean ± SE. Different capital letters (A–C) indicate significant differences within different temperature (*p* ≤ 0.05), and different lower-case letters (a–f) indicate significant differences within different time (*p* ≤ 0.05).

The water migration is closely related to the oil uptake and quality of fried food [28]. The dynamic changes in the water content of French fries with different frying temperature are shown in Figure 2. With increasing frying time, the water content of all samples showed a trend of decreasing dramatically and gradually flattening, and they reached equilibrium water contents (M_e_) in 9, 10, 10, and 11 min, respectively. Zhang, Zhang, Fan, Li, and Fan [29] reported that the water content of fried chips decreased from 82% to 2% after frying for 6 min, and there was no significant difference between other samples. The changes in water content with frying time indicated that water loss of fries during frying was divided into three stages: (1) at the beginning of frying process, the heat in the frying oil was transferred to the surface of the food in the form of convection, and at this time, there was no water evaporation. When the surface temperature reached the boiling point of water, the water migrated from the fries to frying medium, which led to the formation of vapor bubbles on the surface of the fries [30]. This phenomenon would continue for a period of time; thus, this stage was called the surface boiling stage. (2) As the frying temperature increased, the fries’ surface formed a crust, and the thickness of the crust gradually increased with the inward movement of water evaporation, resulting in that the water evaporation rate slowed down; thus, this stage was the rate reduction stage [9]. (3) When the water evaporation rate slowed down to the point where almost no bubbles were formed on the surface of fries, the bubble ending stage was entered, and the water loss reached equilibrium at this stage.

The water content of fries decreased with the increasing frying temperature when the time was below 7 min, while the water content of other fries had no significant difference (*p* > 0.05). The changes in the water content of fries with different frying temperatures could be explained in such a way that, as the frying temperature increased, the surface boiling stage accelerated, which increased the water loss of the fries.

#### 3.2.2. Water Loss Kinetic Modeling

**Figure 3:**
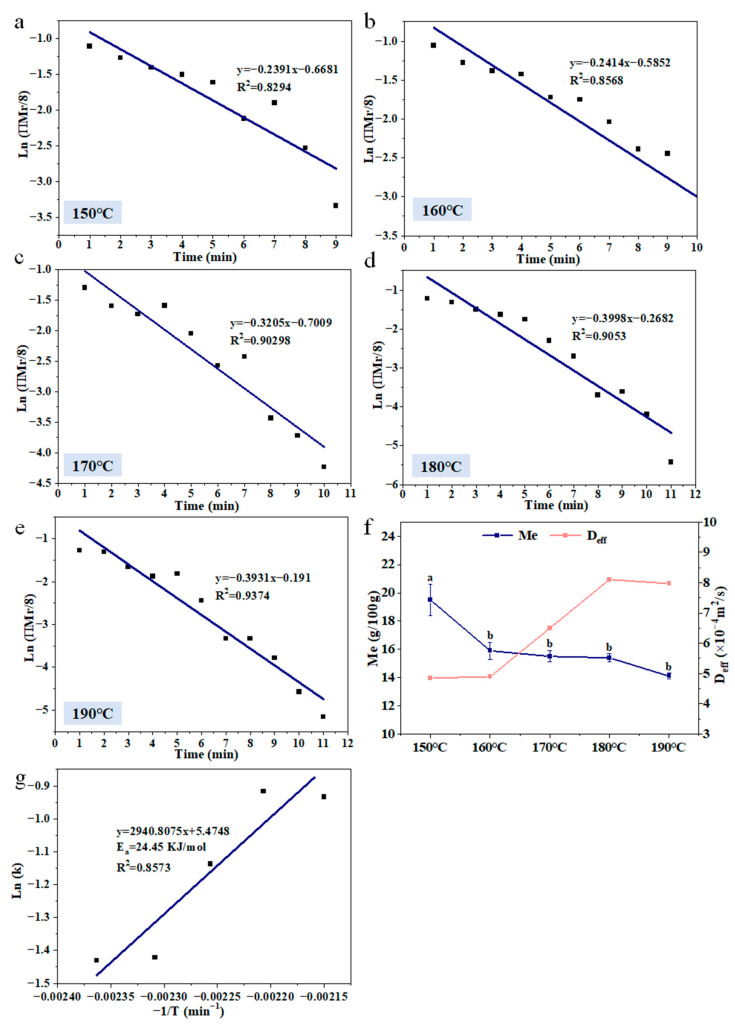
The modeling of water loss kinetics (**a**–**e**), kinetic parameters (**f**), and activation energy of water loss (**g**) in French fries. The results are the mean ± SE. Different letters (a,b) indicate significant differences (*p* ≤ 0.05).

The changes in water loss rate (k_w_), R^2^, D_eff_, and M_e_ of fries with different temperatures and the E_a_ value of water loss are shown in Figure 3. The k_w_ values were obtained from the slopes of fit curves of Ln (ПMr/8) vs. time (Figure 3). The D_eff_ is the water effective diffusivity, which was calculated by k values. The activation energy was used to define the energy barrier that needed to be overcome for a chemical reaction to occur. With frying temperature increased, the k_w_, R^2^, and D_eff_ values of French fries, respectively, increased from 0.2391 to 0.3931; 0.8294 to 0.9371; 4.8505 × 10^−4^ to 7.9736 × 10^−4^, and the Me decreased. The activation energy of water loss in fries was 24.45 KJ/mol, which indicated that a large amount of energy was used for the water evaporation of fries during frying. Oladejo et al. [18] also reported that the k_w_ and D_eff_ values of fried sweet potatoes increased with increasing frying temperature, which indicated that the water evaporation rate and water diffusion rate of French fries increased with increasing frying temperature.

### 3.3. Oil Uptake and Kinetic Modeling of French Fries

#### 3.3.1. Oil Content

**Figure 4:**
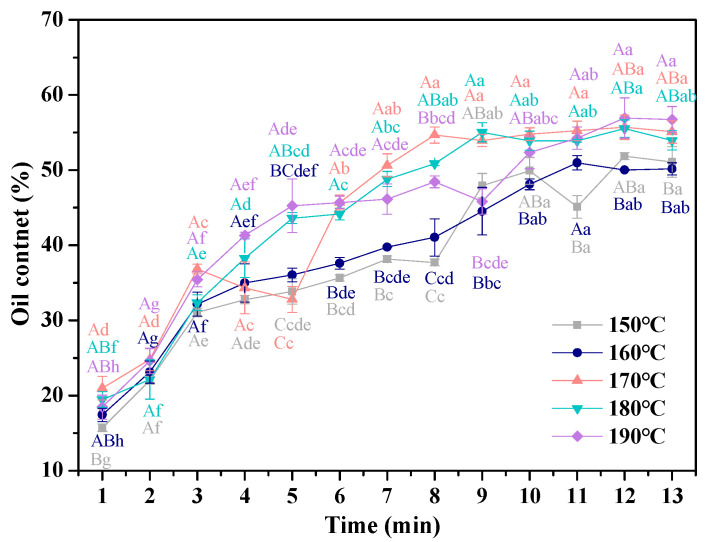
Effect of frying temperatures and times on the oil content in French fries. The results are the mean ± SE. Different capital letters (A–C) indicate significant differences within different temperature (*p* ≤ 0.05), and different lower-case letters (a–f) indicate significant differences within different times (*p* ≤ 0.05).

The dynamic changes in the oil content of French fries fried with different frying temperatures are exhibited in Figure 4. As the frying times prolonged, the oil content of the fries increased from 7.8% to 45–55% and showed a trend of first increasing and then reaching a plateau. Oil absorption of fried foods during frying is a complex process, and there are three possible mechanisms to explain the oil absorption of fried foods, namely water replacement mechanism, condensation effect, and surface-active agents [31]. The water replacement mechanism explained that the escape of water created water vapor channels, holes, and cracks in the fries, which oil could penetrate [8]. After frying, the fries were taken out of fryer, and the pressure of the holes on the fries’ surface reduced, leading to the oil that adhered to the surface being sucked into the pores, which was described by the condensation effect [32]. The third mechanism existed in foods that fried for a long time: as the frying time prolonged, the interfacial tension between the fries’ surface and oil decreased, due to the hydrolysis of oil, resulting in oil that easily penetrated into the fries [33]. The changes of the oil content with the increase of frying time could be explained by the fact that the pores of fries left by the water evaporation increased, which provided enough spaces for oil penetration [34], leading to the oil content increase. As no water evaporated from the fries, the oil uptake behavior of the oil gradually stopped.

With the increasing frying temperature, the oil content of French fries first increased and then decreased, which could be explained by the fact that the pressure in the water channels increased, which was caused by water evaporation increase [35], which prevented the oil from penetrating.

#### 3.3.2. Oil Uptake Kinetic Modeling

**Figure 5:**
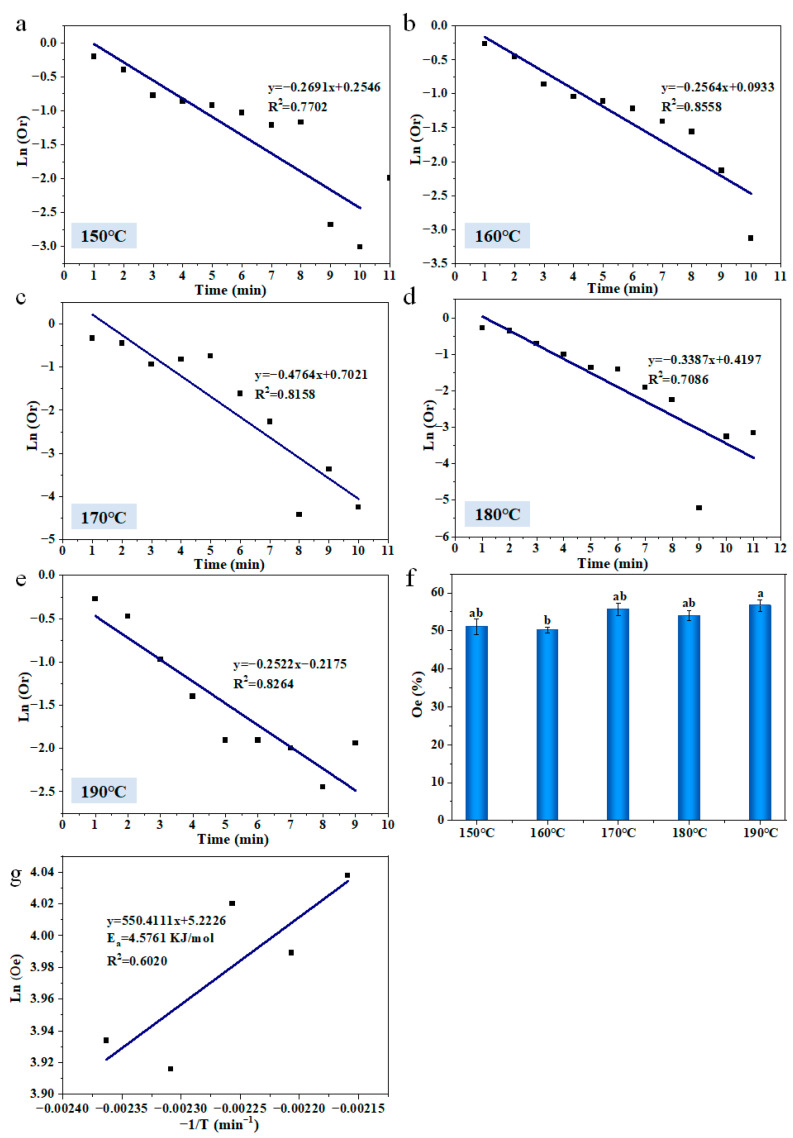
The modeling of oil uptake kinetics (**a**–**e**), kinetic parameters (**f**), and activation energy of oil absorption (**g**) in French fries. The results are the mean ± SE. Different letters (a,b) indicate significant differences (*p* ≤ 0.05).

The fit curves of the Ln (O_r_) vs. time, oil uptake rate (k_o_), and equilibrium oil content (O_e_) values of French fries with different frying temperatures and E_a_ values of oil uptake are displayed in Figure 5. The k_o_ values of fries were obtained from the slopes of fit curves. With the increasing temperature, the oil uptake rate (k_o_) and O_e_ showed a trend of first increasing and then decreasing, and the maximum value was obtained at 170 °C. The k_o_ values of fries fried at 150, 160, and 170 °C were higher than the k_w_, while the k_o_ values of samples fried at 180 and 190 °C were lower than the k_w_. The changes of k_o_ and O_e_ values in samples with different frying temperature were attributed to the fact that, as the frying temperature increased, the bubbles covering the surface of fries formed a barrier [36], preventing the oil from contacting the surface of fries. Additionally, the higher the frying temperature, the denser the bubbles covering the surface of fries, and the more difficult it is for the oil to contact the fries’ surface. The activation energy of the oil absorption in fries was 4.58 KJ/mol, which was lower than the E_a_ of water loss, implying that the fries required less energy to absorb oil than water evaporation.

### 3.4. Color

The golden color corresponds to higher L* and b* values and lower a* values, which is an important quality parameter influencing consumers’ choice of French fries [37]. The ΔE values were calculated by Equation (11), which reflected the color difference between unfried potato strips and French fries [7]. The BI values that characterized the degree of browning were calculated by Equation (12) [38].

**Figure 6:**
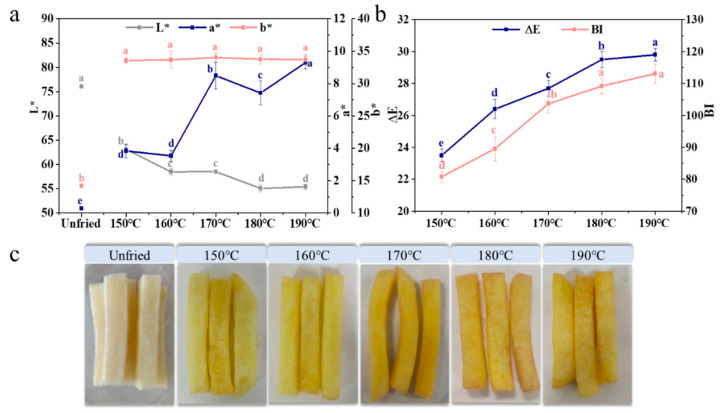
Effect of frying temperature on the color (**a**,**b**) and appearance (**c**) of French fries. Different letters (a–e) indicate significant differences (*p* ≤ 0.05).

The changes in the colors of the French fries were reflected by L *, a*, b*, ΔE, BI values, and appearance (Figure 6). With the increasing frying temperature, the L* values of fries showed a trend of decreasing, and the a* values first increased and then decreased. There was no significant difference in the b* values of fries treated with different frying temperatures (*p* > 0.05). The ΔE and BI values of fries showed an opposite trend with L*. As the ΔE values > 5, the color difference between the fries was visible to the naked eye [39]. The ΔE >5 was observed between the samples at 150 °C and samples fried at 180 and 190 °C, which indicated that the color difference was visible to the naked eye. The color changing from light yellow, golden yellow to deep yellow was observed by the visual appearance of the fries. The decreasing L* and increasing a*, ΔE, and BI values could be explained by the fact that, with the rising frying temperature, the starch gelatinization degree increased [40], which exposed the reducing terminals in the starch, resulting in the increasing Maillard reaction of fries. In addition, the water content of fries played a key role in the changes of the L* values. As the frying temperature increased, the water content of fries decreased (Figure 2), which reduced the light reflection on the fries’ surface [41].

### 3.5. Texture

**Figure 7:**
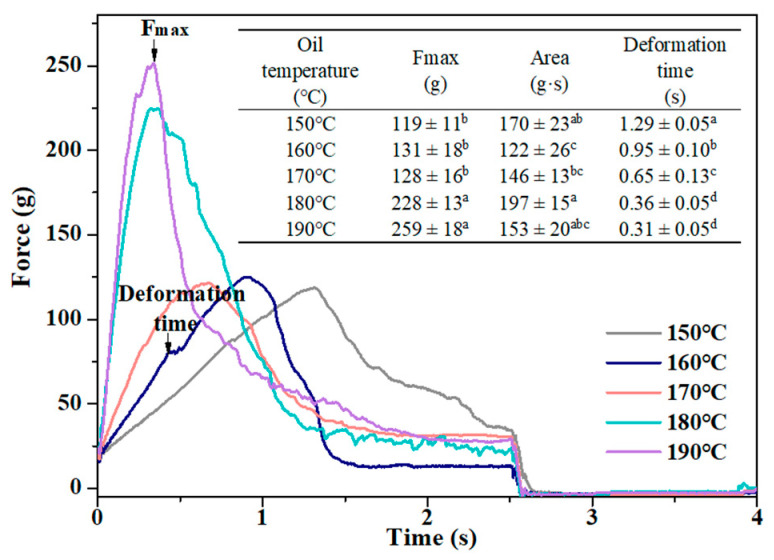
Effect of frying temperature on the texture of French fries. Different letters (a–d) indicate significant differences (*p* ≤ 0.05).

The crispy texture is one of the features of French fries that attracts consumers. The crust hardness, overall hardness, and crispness of fries could be reflected by the F_max_, area, and deformation time values, which were obtained from the force–time curves (Figure 7) [8]. The curves of all samples showed a trend of rising first and then falling, which indicated that the fries had a hard crust and moisture core. With the rising frying temperature, the F_max_ of fries increased, and the area values first decreased and then increased. The deformation time exhibited an opposite trend with the F_max_ values. The increase in the crust and overall hardness of fries were attributed to the increasing water loss [42] and crust thickness [43]. The water evaporated intensively at high frying temperatures, which promoted the evaporation of the water inward, leading to an increase of crust thickness [43]. Liu, Tian, Hu, Yu, and Fan [44] also reported that, the thicker the crust, the greater F_max_ required to puncture. The changes in crispness were closely related to the porous structure of fries [11]. The empty holes and cracks in the fries caused by water vapor escape increased with the increasing frying time, which gave the fries a crispier texture.

### 3.6. Sensory Evaluation

The sensory evaluation of fries with different frying temperature is shown in Table 1. With increasing temperature, the color and overall acceptability of the fries first significantly increased and then decreased (*p* ≤ 0.05), and the best scores were obtained at 170 °C. The crispness of the fries increased as the frying temperature increased, and there were significant differences between samples fried at 150 °C and other samples (*p* ≤ 0.05). There were no significant differences in the aroma and oiliness of all fries (*p* > 0.05). During frying, a series of complex reactions occurred in the fries, such as water evaporation [45], changes in microstructure [46], starch gelatinization [47], and Maillard reaction [48], which endowed the fries with a golden color and crispy texture. With the increasing temperature, these complex reactions accelerated, resulting in the improvement of color, texture, and overall acceptability scores.

## 4. Conclusions

With the increase of frying temperature, the heat transferred from the frying medium to fries’ center was hindered. The dynamic changes in water and oil content showed that the water evaporation and oil uptake increased and then reached a plateau, with the frying time prolonged. The modeling of water loss kinetics and oil uptake kinetics of fries during frying was established by the fitting curves. With increasing frying temperatures, the Kw of fries increased, meanwhile the k0 of fries first increased, then decreased, and showed the maximum values at 170 °C. The a*, ΔE, browning degree, and texture improved with increasing frying temperature. The golden color could be observed by the appearance of the samples fried at 170 °C. The sensory evaluation showed that the color, texture, and overall acceptability of samples fried at 170 °C was highest. Therefore, the changes in the quality and oil uptake of fries were owed to the non-enzymic browning and the formation of crust and pores, which was closely correlated with heat transfer and water evaporation. This study provided new insights for the mechanism of oil absorption in fried food and made it possible for the industrial production of low-oil fried foods with excellent quality.

## Figures and Tables

**Table 1:** Effect of frying temperature on the sensory evaluation of French fries.

Temperature (°C)	Color	Crispness	Aroma	Oiliness	Overall Acceptability
150	6.03 ± 0.63 ^cd^	5.73 ± 0.79 ^b^	5.83 ± 1.30 ^a^	6.73 ± 0.92 ^a^	5.96 ± 1.05 ^b^
160	5.680 ± 0.85 ^d^	6.58 ± 0.84 ^ab^	6.28 ± 1.07 ^a^	6.86 ± 1.08 ^a^	6.45 ± 0.96 ^ab^
170	7.45 ± 0.60 ^a^	7.33 ± 0.72 ^a^	7.10 ± 1.05 ^a^	6.63 ± 1.08 ^a^	7.25 ± 0.79 ^a^
180	6.91 ± 0.29 ^ab^	7.03 ± 0.66 ^a^	7.05 ± 1.09 ^a^	6.70 ± 0.86 ^a^	7.20 ± 1.09 ^ab^
190	6.62 ± 0.68 ^bc^	7.38 ± 0.43 ^a^	7.18 ± 1.17 ^a^	6.83 ± 0.82 ^a^	7.10 ± 1.10 ^ab^

Different letters (a–d) indicate significant differences (*p* ≤ 0.05).

## Data Availability

The data presented in this study are available in the article.
